# MR-Guided Adaptive Radiotherapy for Head and Neck Cancer: Prospective Evaluation of Migration and Anatomical Changes of the Major Salivary Glands

**DOI:** 10.3390/cancers13215404

**Published:** 2021-10-28

**Authors:** Janita E. van Timmeren, Madalyne Chamberlain, Marta Bogowicz, Stefanie Ehrbar, Riccardo Dal Bello, Helena Garcia Schüler, Jérôme Krayenbuehl, Lotte Wilke, Nicolaus Andratschke, Matthias Guckenberger, Stephanie Tanadini-Lang, Panagiotis Balermpas

**Affiliations:** Department of Radiation Oncology, University Hospital Zurich and University of Zurich, 8091 Zurich, Switzerland; madalyne.chamberlain@usz.ch (M.C.); marta.bogowicz@maastro.nl (M.B.); stefanie.ehrbar@usz.ch (S.E.); riccardo.dalbello@usz.ch (R.D.B.); helena.garciaschueler@usz.ch (H.G.S.); jerome.krayenbuehl@usz.ch (J.K.); lotte.wilke@usz.ch (L.W.); nicolaus.andratschke@usz.ch (N.A.); matthias.guckenberger@usz.ch (M.G.); stephanie.tanadini-lang@usz.ch (S.T.-L.); panagiotis.balermpas@usz.ch (P.B.)

**Keywords:** head and neck cancer, image-guided radiotherapy, MRI-linac, adaptive radiotherapy, parotid glands, submandibular glands

## Abstract

**Simple Summary:**

During radiotherapy of head and neck cancer patients, the radiation dose for the major salivary glands (parotids and submandibular glands) should be kept as low as possible to reduce toxicity risks. Nevertheless, volume changes and positional shifts of salivary glands during treatment may result in an increased dose, so adapting the treatment plan is recommended. While previous studies primarily used one or two CTs or CBCTs for imaging during treatment, frequent MRI allows for improved soft tissue contrast and more exact contour adaptation without an additional imaging dose. In this study, twelve patients were treated with MRI-guided radiotherapy with weekly offline adaptations. Significant parotid- and submandibular gland shrinkage, as well as a medial shift of the parotids, were observed. These results support the rationale of MR-guided radiotherapy for head and neck cancer, emphasizing the relevance of future clinical evaluation of toxicity to optimize the benefits of MRI-guided radiotherapy.

**Abstract:**

The aim of this study was to quantify anatomical changes of parotids and submandibular glands and evaluate potential dosimetric advantages during weekly adaptive MR-guided radiotherapy (MRgRT) for the definitive treatment of head and neck cancer (HNC). The data and plans of 12 patients treated with bilateral intensity-modulated radiotherapy for HNC using MR-linac, with weekly offline adaptations, were prospectively evaluated. The positional and volumetric changes of the salivary glands were analyzed by manual segmentation in weekly MRI images and the dosimetric impact of these anatomical changes on the adapted treatment plans was assessed. The mean volume change in parotid and submandibular gland volume was −31.9% (*p* < 0.0001) and −29.7% (*p* < 0.0001) after five weeks, respectively. The volume change was significantly correlated with the cumulative dose for the respective gland at the time of volume measurement. Inter-parotid distance changed by −5.4% (6.5 mm) on average after five weeks (*p* = 0.0005). The distance became significantly smaller only in the left-right direction. The inter-submandibular gland distance changed by 0.7 mm (*p* = 0.38). This study demonstrated significant changes in salivary gland volumes and position following daily MR guidance and weekly plan adaptation. Ongoing clinical trials will provide data on the clinical impact of these changes and novel MR-based adaptation strategies.

## 1. Introduction

Xerostomia is one of the most frequently observed kinds of toxicity after radiotherapy of head and neck cancer (HNC), which significantly impacts the quality of life [1]. To reduce the risk of xerostomia, it is recommended to minimize the radiation dose to the salivary glands, including the parotid glands and the submandibular glands. Nevertheless, numerous studies have shown that significant anatomical changes occur during radiotherapy for HNC (Table S1), caused by weight loss, swelling, edema, sinus filling, response to treatment or setup changes, e.g., when the patient is in pain or soft tissue in the neck changes slightly in position. Volumetric and positional changes of the salivary glands might cause an increase in dose during radiotherapy. To account for geometrical changes during treatment, image-guided adaptive radiotherapy (IGART) has been recently proposed and evaluated to improve target coverage, organ at risk (OAR) sparing and minimize the risk of toxicity [2,3,4]. Nevertheless, imaging is mostly based on computer tomography (CT) and typically one replan is made mid-treatment to account for large anatomical changes [5], whereas patients might benefit more from weekly replanning [6]. The development of a hybrid magnetic resonance imaging linear accelerator (MR-linac) is the first technique that allows for IGART with frequent and/or online treatment plan adaptation, which is made possible by improved visualization of tumors and OARs and is not associated with an additional imaging dose [7,8,9,10,11]. Following the advent of MR-guided radiotherapy (MRgRT) and its subsequent utilization for the treatment of HNC [4], some novel prospective and retrospective studies have been initiated with the purpose of investigating the clinical impact of MRgRT on xerostomia [12,13]. While trials with clinical endpoints are ongoing, the aim of this study was to evaluate the volumetric and positional changes of the parotid glands and submandibular glands during radiotherapy using weekly acquired MRI images and subsequently evaluate the potential benefit of weekly adaptation of the dose to the salivary glands.

## 2. Materials and Methods

### 2.1. Patients

The patients included in this analysis presented with newly diagnosed, histologically confirmed squamous cell carcinoma (SCC) of the oropharynx, hypopharynx or larynx in the UICC stages II–IVB. Consecutive patients without contraindications for MRI (e.g., claustrophobia, metal implants, tattoos, pacemakers) and indications for bilateral definitive concomitant cisplatin-based chemoradiotherapy with curative intent were included. MRgRT was performed with a ViewRay MRIdian Linac (Viewray, Inc., Oakwood Village, OH, USA) [14]. All the patients signed informed consent forms for further use of their data. All the data were prospectively collected and analyzed. Collection and use of the data were approved by the local ethics committee (approval numbers 2018-01794 and 2019-00993).

### 2.2. Pretreatment

One week prior to treatment, an ^18^F-FDG-PET/CT scan and MRI were acquired in the treatment position and coregistered. A tabletop overlay was used on the CT scanner to mimic the situation on an MR-linac. All the MRI scans were acquired with a dedicated head and neck coil (ViewRay Inc., Oakwood Village, CA, USA) using the balanced steady-state free precession (bSSFP) sequence, resulting in the T2/T1-weighted contrast. The images were reconstructed into isotropic voxels of 1.5 mm^3^. For simulation and during treatment, the patient was immobilized with a custom 5-point thermoplastic mask and cushion (CIVCO Radiotherapy, Coralville, IA, USA). Contouring was performed on the MRI by an experienced radiation oncologist specialized in radiotherapy for HNC. Target volume definitions were performed according to the international guidelines [15,16,17]. The macroscopic tumor and the involved nodes (according to the FDG uptake) were defined as the gross tumor volume (GTV). A sequential boost approach was used for all the cases. The high-risk clinical target volume (CTV) was defined as the GTV plus a 5 mm 3D margin (CTV3). The intermediate-risk CTV was defined as the GTV plus 10 mm and anatomically involved lymph node levels (CTV2). Elective CTV included bilateral cervical node levels (CTV1). The narrowest recommended safety margins were applied: the planning target volume (PTV) was defined as the corresponding CTV plus 3 mm [15,18]. It should be noted that nodal level Ib including the whole submandibular gland was included in the elective CTV in case of involvement of ipsilateral level II.

### 2.3. Treatment Planning 

An initial treatment plan was made on a ViewRay treatment planning system (TPS) and a backup plan for a conventional linac was made using Eclipse TPS (Varian Medical Systems, Palo Alto, CA, USA). 

Initial treatments were planned with 9–19 beams using modulated 3D inversed planning with the maximum number of segments set to 115. Treatment delivery time below 20 min was intended. All the patients were planned to have 35 fractions of 2 Gy, resulting in the total planned dose of 70 Gy to the macroscopic tumor (PTV3), 60 Gy to the extended tumor region and the involved nodal levels (PTV2) and 50 Gy for elective nodal irradiation (PTV1), which is the standard of care for locally advanced HNSCC [19,20]. Parotid-sparing intensity-modulated radiotherapy (IMRT) aimed to keep the mean dose of at least the contralateral parotids under 24 Gy [2,21]. All the other dose constraints were according to the DAHANCA 2020 radiotherapy guidelines [22].

Dose was calculated with the Monte Carlo (MC) algorithm that takes into account the magnetic field and a grid size of 3.0 mm. Quality assurance (QA) prior to treatment consisted of dose verification using the RadCalc QA software and verification of plan complexity. Delta 4 measurements were performed in case either of the verifications exceeded the acceptance limits but always prior to the delivery of the first treatment plan of each series.

### 2.4. Treatment

All the patients were treated using an MRIdian hybrid linear accelerator (ViewRay Inc., Oakwood Village, CA, USA) with step-and-shoot IMRT using six megavoltage (MV) flattening filter-free (FFF) photon beams with a dose rate of 600 MU/min. Gating during treatment was applied to allow the beam to stop in case of large motion (e.g., coughing, swallowing). 

### 2.5. Imaging during Treatment

Patient setup was evaluated using in-room MRI on a daily basis. A rigid registration with the planning MRI was performed and the patient position was adjusted accordingly in three dimensions. 

### 2.6. Weekly Adaptation

Once a week, the treatment plan was adapted offline, with a maximum of six times during the treatment course. To this extent, the MRI-of-the-day was registered to the MRI of the treatment plan of the week before using deformable image registration (DIR) available in ViewRay TPS to transfer the contours. The target and OAR contours, including salivary gland contours, were then manually adjusted by one out of the two experienced radiation oncologists that were involved in this study. Next, the treatment plan was reoptimized using full IMRT plan optimization. In most cases, the beam setup was not changed. The initial treatment plan was used as a reference and dose volume histograms (DVHs) were compared to ensure similar plan quality. 

**Figure 1:**
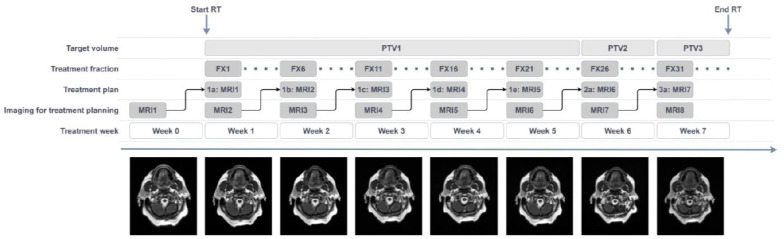
Treatment schedule for weekly MRI-guided offline adaptive radiotherapy in the MARTHA trial. The numbering of the treatment plans (1a–3a) and the number of the MRIs (MRI1–MRI8) is used throughout the manuscript. RT = radiotherapy, PTV = planning target volume, MRI = magnetic resonance imaging, FX = fraction.

The treatment schedule is presented in Figure 1, which also indicates the numbering of the MRIs and the treatment plans that is used throughout the manuscript.

### 2.7. Evaluation in MIM

Weekly MRI images and the corresponding dose distributions (RTDOSE) and contours (RTSTRUCT) were exported from the ViewRay system and imported into the commercial software package MIM Maestro (MIM Software Inc., Beachwood, OH, USA) for further evaluation as outlined below. The salivary glands (parotids and submandibular glands) on the side of the primary tumor were defined as the ipsilateral glands, and on the opposite side—as the contralateral glands. In case of a symmetric bilateral treatment of a centrally located tumor without clear lateralization, the term “central” was used. 

### 2.8. Anatomical Changes

The volume of all the four major salivary glands was evaluated based on the weekly acquired MR images. The relative volume change was calculated with the MRI acquired immediately before delivery of the first fraction as reference (MRI2) as follows: (MRIx − MRI2)/MRI2. Additionally, linear regression was performed to estimate the changes in volume over the course of treatment. 

To investigate a potential migration of salivary glands during the course of fractionated radiotherapy, the geometrical centers were extracted in MIM to calculate the inter-gland distance. Besides the inter-gland distance, the migration of the glands was studied by calculating the distance between a gland and the geometrical center of the brainstem. 

Additionally, the relationship between the cumulative dose for the salivary glands at the time of volume/position measurement and the respective anatomical changes was investigated using linear regression. The Wilcoxon signed-rank test was used to assess whether changes with respect to the start of radiotherapy were statistically significant; *p*-values were based on two-tailed significance unless otherwise specified, and *p*-values below 0.05 were considered significant. The statistical analysis was performed in R (version 4.0.2) [23].

Finally, for the ipsilateral parotid glands, it was investigated whether a reduction in the GTV volume resulted in a reduction in the mean parotid dose using Spearman’s rank correlation coefficient.

### 2.9. Weight Loss

The patients’ weight was recorded weekly during treatment. The relationship between weight loss and the glands’ anatomical changes was investigated using linear regression of the relative change with respect to week 0 (MRI1). Furthermore, the neck half-thickness at the level of the C2 vertebral body was measured on MRI1 and MRI7 [24]. The relationship between the relative neck half-thickness and anatomical changes was investigated in the same fashion as for weight loss. 

### 2.10. Dose Accumulation

**Figure 2:**
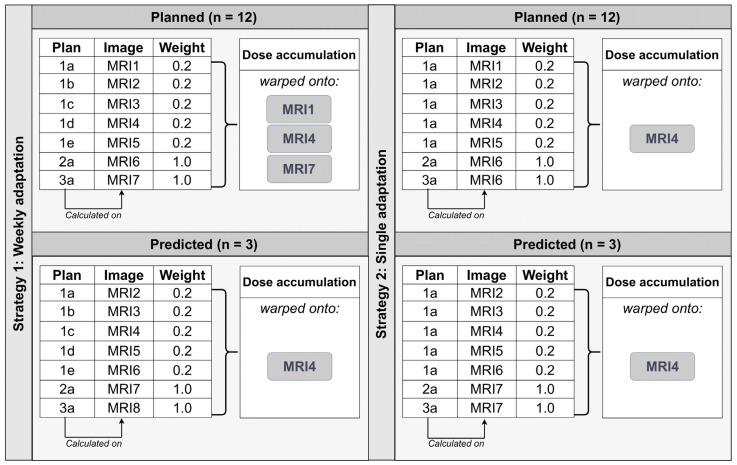
Overview of dose accumulation. Top left: cumulative planned dose of weekly adaptation using MRI1, MRI4 or MRI7 as the reference image. Top right: cumulative planned dose of one single adaptation obtained by recalculating plan 1a on MRI1–MRI5. Bottom left: cumulative predicted dose of weekly adaptation calculated for a subset of three patients obtained by recalculating each weekly plan on the MRI of one week later. Bottom right: cumulative predicted dose of one single adaptation calculated for a subset of three patients obtained by recalculating plan 1a on the images of one week later compared to the cumulative planned dose.

Dose accumulation using DIR with Reg Reveal and Reg Refine in MIM was used to evaluate the cumulative dose for the salivary glands in order to consider the dosimetric impact of anatomical changes during the course of radiotherapy. The doses were weighted according to the number of treatment plans in each series. The accumulated dose was mapped onto the mid-treatment image (MRI4) for all the patients. A comparison was made with the accumulated dose mapped on MRI1 and MRI7 (Figure 2, top left). The DIR-based cumulative dose was also compared to the sum of the mean dose for the salivary glands measured at each weekly radiotherapy plan, normalized according to the number of MRIs.

Two adaptive radiotherapy strategies were evaluated: a strategy with weekly plan adaptation and a strategy of only one single adaptation after week 5 for CTV2 and CTV3, which is the current routine clinical practice in our and other institutions. The latter strategy was simulated by recalculating the dose of treatment plan 1a on all of the MRI1–MRI5. These doses, as well as the dose of plan 2a and the dose of plan 3a recalculated on MRI6 were subjected to DIR to warp them onto MRI4. Next, a weighted summation of these doses was performed (Figure 2, top right). 

Additionally, since the treatment protocol assumes that the anatomy remains stable for one week (i.e., a one week time slot is reserved for the offline adaptation), we evaluated the difference between the cumulative planned dose (dose accumulation of the planned treatment plans) and the cumulative predicted dose (dose accumulation of the treatment plans recalculated on the image of one week later) for the three patients. For instance, to calculate the cumulative predicted dose of weekly adaptation, treatment plan 1a was recalculated on MRI2, 1b was recalculated on MRI3, etc. (Figure 2, bottom left). 

Dose recalculations were performed in ViewRay TPS. The recalculated doses were exported from ViewRay and imported in MIM, in which dose accumulation was performed as described before. 

Besides visual inspection of the DIR, the quality of the DIR-based dose accumulation was assessed by calculating the Dice similarity coefficient (DSC) for the salivary glands where the manual contours were considered the ground truth. MRI4 was used as reference since this was the MRI onto which the accumulated dose was mapped in all the abovementioned situations. 

### 2.11. Modeling 

Stepwise regression was performed to evaluate which parameters contributed most to the dose difference in the D_mean_ that was found between weekly adaptation and one single adaptation for the parotid glands: parotid V_30Gy_ at MRI1, parotid D_mean_ at MRI1, parotid volume at MRI1, parotid overlap and PTV1 on MRI1, parotid volume difference between MRI3 and MRI2, parotid–brainstem distance difference between MRI3 and MRI2, patient weight, age and gender. Each parotid was considered to be an individual subject. The function regsubsets of the R package leaps was used, and the maximum number of variables was set to 2 due to the limited dataset size and the desire for a simple model [25].

## 3. Results

### 3.1. Treatment

At the time of evaluation, twelve patients were included. Table 1 shows the patient and tumor characteristics of the studied cohort. All the patients received at least 33/35 fractions on the MR-linac, and 7/12 patients received all the 35 fractions on the MR-linac. A total of 413/420 fractions were delivered on the MR-linac; the remaining seven fractions were delivered on the conventional linac due to technical problems with the MR-linac. The time used for recontouring was approximately one hour, whereas half a day was re-served for this task, as well as for offline replanning. The exact times were not recorded. The maximum allowed time to complete the process of reoptimization including all the required QAs was five days, and this was always respected.

On the initial treatment plan, the D_mean_ of the ipsilateral parotid glands was 27.9 ± 10.6 Gy (mean ± SD), whereas this was 17.6 ± 3.2 Gy for the contralateral parotid glands, 48.8 ± 6.1 Gy for the ipsilateral submandibular glands and 38.9 ± 11.8 Gy for the contralateral submandibular glands (Table 1). The average volumetric overlap of all the glands with PTV1 was 5.4% for the parotid glands and 64.7% for the submandibular glands (Table 1).

### 3.2. Volume Changes

**Figure 3:**
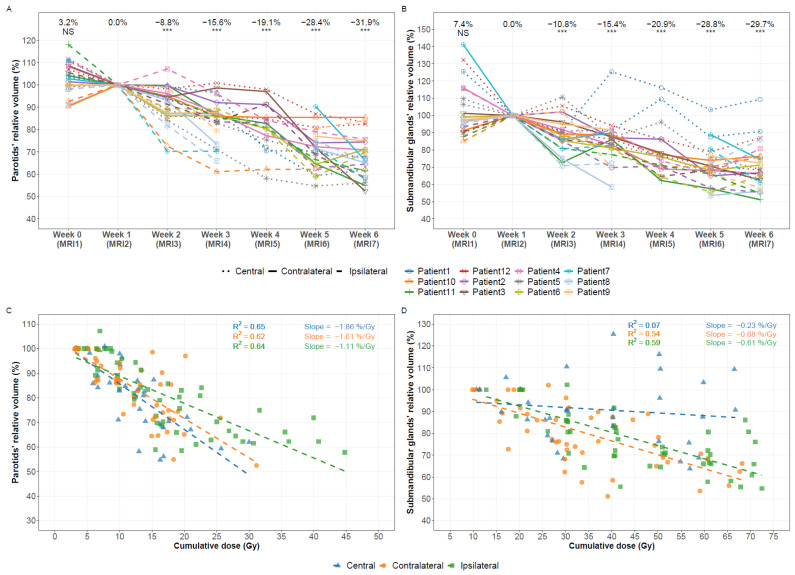
Volume changes of the salivary glands during treatment and the relationship of volume change with the cumulative dose for the respective gland. MRI2 acquired immediately before delivery of the first fraction was set as reference. (**A**) Volume changes of the parotid glands. (**B**) Volume changes of the submandibular glands. (**C**) Volume change of the parotids vs. the cumulative dose for the parotids at the time of volume measurement. (**D**) Volume change of the submandibular glands vs. the cumulative dose for the submandibular glands at the time of volume measurement. In case of a symmetric bilateral treatment of a centrally located tumor without clear lateralization, the term “central” was used to indicate the location of the salivary glands. NS: not significant, *** *p* < 0.001.

The mean (range) change in the parotid volume was −8.8% (−29.7–7.2) after one week of treatment (*p* < 0.0001) and −31.9% (−47.5–−14.5) after five weeks (*p* < 0.0001) (Figure 3A), corresponding to −8.2 mL (−13.4–−3.7). The average volume reduction was not significantly different between the ipsilateral and contralateral parotids (33.3% vs. 31.6%, *p* = 0.73). 

For the submandibular glands, the volume change was −10.8% (−29.1–10.6) after one week (*p* = 0.0003) and −29.7% (−48.8–9.3) after five weeks (*p* < 0.0001) (Figure 3B), corresponding to −2.5 mL (−6.6–0.6). For patient 1, the volume of the right submandibular gland increased by 1.5 mL between week 1 and week 3, but no clear explanation for this relatively large (25%) increase could be found. Again, comparable volume reductions were observed for the ipsilateral and contralateral submandibular glands (33.0% vs. 33.6%, *p* = 0.65). 

The volume change at the end of treatment between the parotids and the submandibular glands was not significantly different (*p* = 0.73). Linear regression showed an average decrease of 0.21 mL/day and 0.07 mL/day for the parotids and submandibular glands, respectively. 

The gland volume change was significantly correlated with the cumulative dose with slopes of −1.1% Gy^−1^ (R^2^ = 0.64) for the ipsilateral parotids and −1.6% Gy^−1^ (R^2^ = 0.62) for the contralateral parotids (Figure 3C). A significant interaction was found in the relationship of volume change and dose for the ipsilateral and central parotids (*p* = 0.011) but not for the ipsilateral and contralateral parotids (*p* = 0.073) and the contralateral and central parotids (*p* = 0.68). The slopes were −0.6% Gy (R^2^ = 0.59) for the ipsilateral submandibular glands and −0.7% Gy^−1^ (R^2^ = 0.54) for the contralateral submandibular glands (Figure 3D). A significant interaction was found for the contralateral and central submandibular glands (*p* = 0.030) and for the ipsilateral and central glands (*p* = 0.0021) but not for the ipsilateral and contralateral ones (*p* = 0.58). A significant interaction was also found in the relationship of volume change and cumulative dose for the parotids and the submandibular glands (*p* = 0.021).

The change in the mean dose for the ipsilateral parotid gland compared to the previous week did not significantly correlate with the change in the GTV volume compared to the previous week (rho = −0.17, *p* = 0.34).

### 3.3. Migration

**Figure 4:**
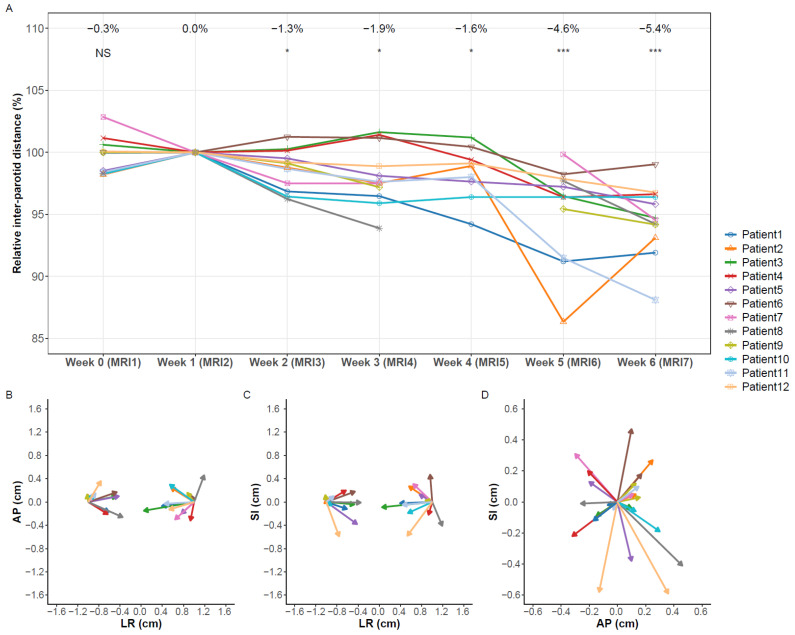
Migration of the parotid glands during radiotherapy. (**A**) Change of the inter-parotid gland distance. (**B**–**D**) Parotid migration in AP/LR, SI/LR and SI/AP. For this representation, the parotid location was corrected with the brainstem location, and then the baseline parotid location was set to (−1,0) for the right parotid and (1,0) for the left parotid. AP = anterior–posterior, LR = left–right, SI = superior–inferior. NS: not significant, * *p* < 0.05, *** *p* < 0.001.

Inter-parotid distance changed on average by −5.4% (−11.9–−1.0%) after five weeks (*p* = 0.0005), corresponding to −6.5 mm (−14.9–−1.0) (Figure 4A). The shift was only significant in the left-right (LR) direction, with a medial shift of the median (range) of 4.0 mm (−1.9–9.5 mm) (*p* = 0.0034) for the left parotid and –3.1 mm (−6.5–0.2 mm) (*p* = 0.0024) for the right parotid (Figure 4B,C). The distance to the brainstem decreased by 1.9 mm and 1.8 mm for the left and right parotids, respectively. 

The inter-submandibular gland distance remained stable with changes < 1 mm (*p* = 0.38) (Figure S1) and was not significant in any direction. The distance to the brainstem decreased by −1.1 mm and 0.2 mm for the left and right submandibular glands, respectively.

### 3.4. Weight Loss 

After 25 fractions, patients lost on average 1.3 kg (range, −9.0–2.8), and the neck half-thickness reduced on average by 2.8 mm (range, −12.3–4.0). The patient weight change showed a weak correlation with the volume change of parotids and submandibular glands, with R^2^ values of 0.08 (*p* < 0.001) and 0.03 (*p* = 0.02), respectively (Figure S2). A significant linear relationship was observed between the parotid volume and the neck half-thickness (*p* < 0.001, R^2^ = 0.42), but not for the submandibular glands (*p* = 0.39). 

Change of the inter-gland distance was significantly correlated to the patient weight change for the parotid glands (*p* = 0.049) but not for the submandibular glands (*p* = 0.65). The neck half-thickness was significantly correlated to the inter-parotid gland distance (*p* < 0.001) but not to the inter-submandibular gland distance (*p* = 0.26). 

### 3.5. Dose Accumulation

#### 3.5.1. Planned vs. Predicted Dose 

Marginal differences between the cumulative planned dose and cumulative predicted doses were observed (DVHs; see Figure S3). On average, the cumulative predicted dose for the parotid glands was 0.5 Gy (1.5%) higher than the cumulative planned dose (range, −0.4 Gy–1.5 Gy). 

#### 3.5.2. Weekly Adaptation vs. Single Adaptation 

**Figure 5:**
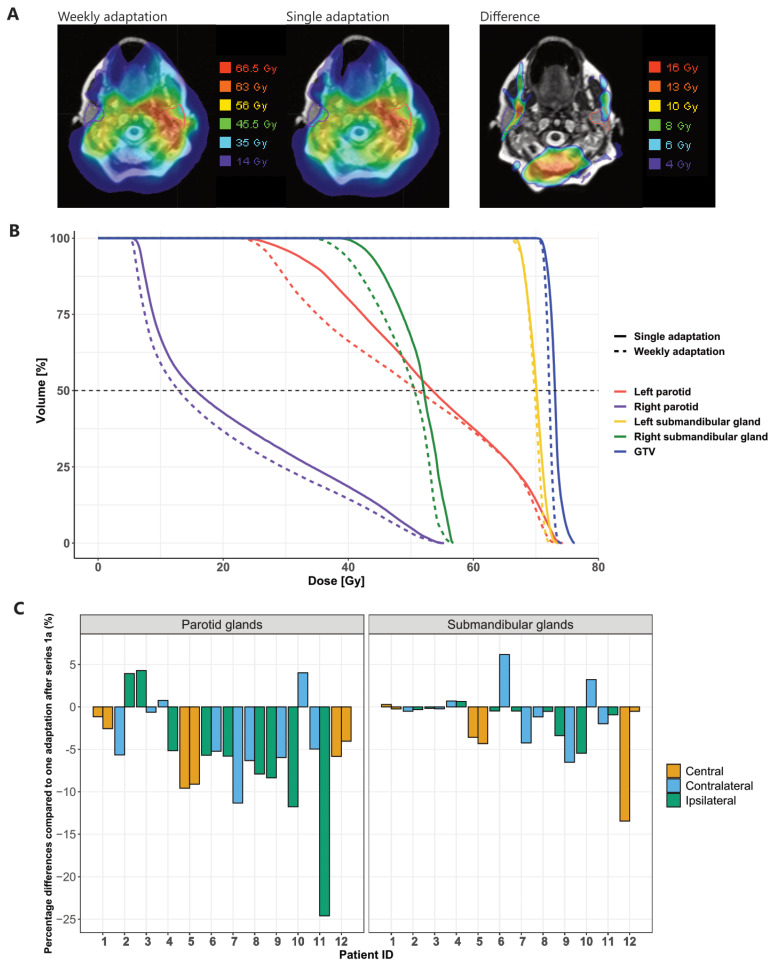
The difference between weekly adaptation and one adaptation at the end of treatment. (**A**) Example of a dose distribution (patient 7) of weekly adaptation (left) or one single adaptation (middle) and the difference between dose distributions (right). (**B**) Corresponding DVHs of the patient in (**A**). (**C**) Dose difference for all the patients for the parotid glands (left) and the submandibular glands (right). Negative values mean that the cumulative planned dose was lower using weekly adaptation. In case of a symmetric bilateral treatment of a centrally located tumor without clear lateraliza-tion, the term “central” was used to indicate the location of the salivary glands.

Figure 5A shows a patient with the cumulative planned dose for weekly adaptation (left) and the cumulative planned dose for the hypothetical situation of one single adaptation, with the corresponding DVHs (Figure 5B). Considering all the patients, the D_mean_ for the parotids was on average 5.4% (1.6 Gy) lower than it would have been with only one single adaptation (Figure 5C), corresponding to 3.9% (0.9 Gy) and 6.8% (2.8 Gy) for the contralateral and ipsilateral parotid glands, respectively. For two ipsilateral parotids glands (patient 10 and 11), the benefit of weekly adaptation was more than 4 Gy. The D_mean_ for the submandibular glands was on average 1.6% (0.9 Gy) lower with weekly adaptation. For one central and one ipsilateral submandibular gland (patient 10 and 12), the benefit of weekly adaptation was more than 4 Gy. For four parotid glands, the mean dose was slightly higher for weekly adaptation compared to the single adaptation, whereas this was the case for five submandibular glands. For the parotid glands, the differences were 3.9% (1.2 Gy), 4.3% (0.9 Gy), 0.8% (0.2 Gy) and 4.0% (0.8 Gy), respectively. For the submandibular glands, the differences were 0.3% (0.2 Gy), 0.7% (0.5 Gy), 0.6% (0.4 Gy), 6.2% (1.9 Gy) and 3.2% (1.2 Gy), respectively. For patient 6, the mean dose for the submandibular glands increased from treatment plan 1a to 1e from 25.5 Gy to 31.3 Gy to ensure coverage of the PTV. All the glands for which the cumulative dose based on single adaptation was lower had an overlap with the PTV, and the submandibular glands of patients 1 and 4 were almost entirely inside the PTV. Therefore, it was not expected that weekly adaptation would necessarily improve the sparing of these glands.

#### 3.5.3. Choice of Reference MRI for Dose Mapping

The differences between the summation of the D_mean_ values and mapping the cumulative planned dose onto MRI1, MRI4 or MRI7 ranged widely from 0.01 Gy to 13.7 Gy (Figure S4). The largest average difference was observed between the doses mapped onto MRI1 and MRI7 (2.5 Gy). A linear summation of the D_mean_ was most comparable to the cumulative planned dose on MRI4 with an average difference of 1.1 Gy. Several patients with large anatomical changes also showed large differences depending on which MRI day the dose was mapped, as shown for patient 7 as an example in Figure S5.

#### 3.5.4. Dice Similarity Coefficient

The average DSC for the parotids was always higher than 0.8 (Figure S6). The average DSC ranged from 0.84 for MRI1 to 0.89 for MRI5 for the parotids, and from 0.81 for MRI1 to 0.85 for MRI5 for the submandibular glands.

### 3.6. Modeling 

Stepwise regression resulted in a simple model with the D_mean_ of the parotid glands at MRI1 (in Gy) and the overlap between the parotid gland and PTV at MRI1 (in %) to predict the dose benefit of weekly adaptation and single adaptation: 5.1 − 0.36 × D_mean_ + 0.21 × Overlap. In other words, a larger initial parotid D_mean_ is expected to result in a larger benefit of weekly adaptation, whereas a larger overlap results in a smaller expected benefit.

## 4. Discussion

We prospectively evaluated the anatomical changes of the salivary glands of the patients treated using MRI-guided radiotherapy with weekly offline adaptation. To the best of our knowledge, only one previous study described the clinical workflow of MR-linac-guided RT for HNC patients and proved clinical feasibility and safety of daily MR-guided ART [12], without special focus on dosimetric analysis of all the four major salivary glands and without reporting any data on gland migration. Marzi et al. also used MR imaging for prospective evaluation, but only examined changes in the parotideal volume and used only one timepoint during treatment after the 10^th^ fraction [26]. All the other studies retrospectively investigated adaptive strategies for HNC using CT or CBCT imaging and at much fewer timepoints compared to this study. The MR-linac allows for online imaging with reduced burden for the patient (offline planning CTs, additional exposure to ionizing radiation) and more frequent adaptation, with improved visibility of the target and OARs [4]. Recently, feasibility of successful planning on the MRIdian platform resulting in high-quality plans for HNC has been demonstrated in a multicenter setting [27]. 

Our study showed that both parotid glands and submandibular glands shrink significantly and continuously during the course of treatment, with both glands losing on average one third of their original volume, which forms the rationale for more frequent adaptation for HNC radiotherapy. As early as one week after the start of treatment, the volume of the salivary glands was significantly reduced. Additionally, significant migration of the parotid glands towards the midline was observed, whereas no migration of the submandibular glands was seen. These observations are in line with previous studies, where parotideal migration was a common observation (Table S1). Some previous studies used MRI to evaluate parotid volume changes during radiotherapy. For instance, a previous study on six patients treated using an MRI-tri-60Co device showed significant volume reduction and center-of-mass shifts of the parotids [27]. Additionally, another study used repeated functional MRI at the 10^th^ treatment fraction and observed significant parotid gland volume reductions [26]. However, all of these studies used X-ray imaging, applied less frequent imaging and adaptation and/or were retrospective. Moreover, none of the previous studies evaluated all the four major glands and migration and volume changes simultaneously.

Weekly adaptation allowed for an average cumulative dose reduction of 5.4% for the parotid glands, as opposed to a schedule with one single adaptation. These results are similar to a recent study that evaluated weekly adaptation on an MR-linac, in which for 12/14 parotid glands, a dosimetric benefit of < 4% was observed, for 2/14 glands—of >4% [12]. In our study, the largest overall benefit of weekly adaptation was observed for the ipsilateral parotid glands, although large patient-to-patient variability was evident. A benefit of 4 Gy was considered clinically relevant, even though the reduction of xerostomia risk could not yet be investigated in this study, and this was observed for four glands of three distinct patients (two parotid and two submandibular glands). This implies that the amount of labor-intensive offline adaptation could be reduced to only those patients that benefit the most from it or only at the moment in treatment that benefits patients most. A previous study evaluated numerous adaptation strategies and showed that patients benefit most from adaptation in the first two weeks of treatment [6]. Daily MR imaging allows for automated decision-making regarding patient-specific adaptation schedules, e.g., a traffic light protocol that warns against adaptation in case or large anatomical changes. This represents an additional benefit of MRgRT for HNC as the exact evaluation of soft tissue and salivary glands in the still most common cone beam CT (CBCT) remains challenging and associated with additional dose exposure for the patient. Criteria for beneficial adaptation should be defined in future studies using modeling of anatomical, clinical, dosimetric and treatment parameters, in which outcome data are available. 

The current adaptation schedule assumes a stable anatomy for a period of one to two weeks (e.g., from the time of MRI acquisition until the last fraction delivered with the respective treatment plan). Despite that, we observed only small differences comparing the planned and predicted cumulative doses. This is in correspondence with the results of Heukelom et al. [28] but in contrast to the results of Brouwer et al. [29]. In the ideal situation, the time required for offline adaptation should be minimized. This could potentially be achieved by experience, the use of more robust deformable image registration methods to reduce the contouring time and automated planning. A further solution that is currently applied in other anatomical localizations could be a daily online adaptation of only critical structures followed by only weight optimization planning for most cases [30]. Advances in software and hardware could make daily online adaptive radiotherapy for HNC more easily implementable in clinical practice [12]. Nevertheless, the clinical benefit of daily adaption has not yet been established. 

Currently, in our imaging protocols, no functional MRI is available. Nevertheless, future access to diffusion-weighted imaging (DWI) could provide additional information on salivary gland tissue changes and for optimization of the adaptation strategy [26]. Moreover, despite the possibilities provided by modern integrated MR imaging, no clear consensus exists on how often an adaptation is necessary, and a balance between expenditure/effort and clinical benefit has yet to be defined. Another unsolved issue is the selection of the optimal dosimetric parameter that should be used for volumetric predictions. Although the D_mean_ is a generally accepted and important parameter known to correlate with clinical consequences, other parameters could also be of importance. Indeed, Broggi et al. retrospectively evaluated data of 87 patients and the corresponding MVCT or CBCT images and demonstrated the significance of the D_mean_ for predicting volumetric differences [31]. However, the volume receiving 40 Gy (V_40Gy_) was also a major predictor. In our study, although V_30Gy_ was included for modeling building, and not V_40Gy_ (highly correlated with Spearman’s rho of 0.92; not shown), only the D_mean_ was included in the final simple model to predict the dosimetric benefit. Furthermore, no correlation was observed between the volume change at the end of treatment and either V_30Gy_ or V_40Gy_ at the baseline (not shown). Nevertheless, these results should be interpreted with caution due to the small sample size. 

This study was an observational evaluation of clinical practice and not a planning study. Therefore, we cannot make any conclusions on what caused any dosimetric benefit. Furthermore, no unambiguous explanation could be found for the glands for which dose accumulation based on single adaptation resulted in a lower dose. The main reason for that was probably the need for PTV coverage arising from anatomical changes. In case of either ensuring sparing of the respective gland or already exceeding the tolerance due to an adjacent tumor, no concerns were brought up during planning for possible slight increases in the mean gland dose. Moreover, there is a possible bias as several planners were involved in this study. Thus, even though dosimetric advantages might be explained partly by volumetric differences and migration of the salivary glands, inter-planner variability across the treatment plans cannot be excluded in our study. An additional limitation of this study is that the cumulative planned dose could not be compared to the hypothetical situation of no adaptations during treatment since treatment plans were used sequentially, and no treatment plan with a simultaneously integrated boost technique was available for comparison. Additionally, the number of patients included here was relatively low to provide robust recommendations. Therefore, further investigations are required to determine the dosimetric benefit of MRgRT for this purpose. Another limitation is uncertainty of deformable image registration. However, the accumulated dose was compared to the mean dose; additionally, visual inspection of the deformations was performed. Furthermore, the DSC analysis showed good performance, with an average DSC of 0.84 for all the salivary glands, complying to the recommendations of AAPM Task Group No. 132 [32]. At the same time, to the best of our knowledge, this is the first study evaluating an MR-linac-based approach for weekly adaptive radiotherapy for HNC including salivary gland monitoring of all the four major glands demonstrating several significant anatomical changes that stress the need for modern adaptive concepts.

## 5. Conclusions

The anatomical changes of salivary glands observed in this study and the cumulative contribution of multiple planners confirm the rationale of adaptive MR-guided radiotherapy for head and neck cancer using a hybrid MR-linac system. Future analysis will include the clinical evaluation of xerostomia, which allows identifying the real-life benefits of MR-guided RT and ways to optimize them for head and neck cancer patients. 

## Figures and Tables

**Table 1:** Patient characteristics. * Two carcinomas, ** UICC TNM 8th edition. In case of a symmetric bilateral treatment of a centrally located tumor without clear lateralization, the term “central” was used. PTV = planning target volume, H = hypopharynx, O = oropharynx, L = larynx, Or = oral cavity, NA = not available.

Patient	Age	Gender	HPV Status	Tumor Site	Tumor Stage	Overall Stage **	Tumor Location	Left Parotid Gland			Right Parotid Gland			Left Submandibular Gland			Right Submandibular Gland		
								Mean Dose	Volume	Overlap PTV1	Mean Dose	Volume	Overlap PTV1	Mean Dose	Volume	Overlap PTV1	Mean Dose	Volume	Overlap PTV1
1	39	male	-	H,O *	T2N3bM0	IVB	Central	17.1 Gy	28.3 mL	8.3%	25.3 Gy	31.1 mL	20.5%	50.3 Gy	9.4 mL	96.8%	50.7 Gy	7.5 mL	97.9%
2	67	male	+	H	T2N1M0	I	Right	16.9 Gy	31.8 mL	0.0%	24.8 Gy	29.5 mL	7.2%	43.9 Gy	11.9 mL	7.7%	51.2 Gy	13.9 mL	99.3%
3	66	female	+	O	T4N2M0	III	Left	16.5 Gy	18.5 mL	4.4%	25.8 Gy	19.3 mL	13.1%	50.3 Gy	6.7 mL	94.2%	50.1 Gy	6.5 mL	73.3%
4	67	female	-	Or	T3N2cM0	IVA	Right	16.3 Gy	20.7 mL	0.1%	17.9 Gy	21.6 mL	1.2%	50.8 Gy	8.0 mL	87.9%	50.5 Gy	7.3 mL	98.6%
5	61	male	-	H	T4N2cM0	IVA	Central	16.8 Gy	18.3 mL	0.6%	17.0 Gy	28.1 mL	0.7%	51.0 Gy	8.6 mL	93.0%	50.7 Gy	7.9 mL	99.1%
6	65	male	NA	L	T3N0M0	III	Left	16.4 Gy	19.0 mL	0.0%	15.3 Gy	21.2 mL	0.2%	32.7 Gy	7.7 mL	32.6%	25.5 Gy	6.4 mL	5.4%
7	56	female	+	O	T1N1M0	I	Left	42.9 Gy	19.0 mL	27.4%	16.2 Gy	19.0 mL	4.0%	50.6 Gy	6.1 mL	100%	48.2 Gy	6.0 mL	51.6%
8	74	male	-	O	T4bN1M0	IVB	Right	18.4 Gy	35.0 mL	2.9%	32.2 Gy	33.4 mL	5.2%	50.2 Gy	7.1 mL	96.9%	50.5 Gy	6.8 mL	100%
9	69	male	-	O	T2N0M0	II	Left	22.2 Gy	31.8 mL	0.5%	16.6 Gy	33.4 mL	0.0%	50.5 Gy	10.9 mL	99.5%	25.6 Gy	9.4 mL	0.1%
10	60	male	+	O	T4N2M0	III	Left	39.7 Gy	25.7 mL	24.0%	16.2 Gy	22.9 mL	0.8%	51.0 Gy	8.8 mL	100%	26.7 Gy	9.1 mL	17.5%
11	57	male	-	O	T4N2bM0	IVA	Right	16.6 Gy	27.2 mL	0.0%	38.3 Gy	29.6 mL	8.2%	29.1 Gy	8.4 mL	0.1%	52.2 Gy	6.4 mL	100%
12	51	male	NA	L	T3N0M0	III	Central	15.6 Gy	41.0 mL	0.0%	16.7 Gy	46.9 mL	0.0%	25.6 Gy	8.4 mL	0.1%	26.8 Gy	14.1 mL	1.7%

## Data Availability

The data presented in this study are available on request from the corresponding author. The data are not publicly available due to the fact that part of the data originated from a prospective ongoing clinical trial (NCT03972072).

## Supplementary Materials

The following are available online at https://www.mdpi.com/article/10.3390/cancers13215404/s1, Figure S1: Migration of the submandibular glands during radiotherapy, Figure S2: Volume and migration versus patient weight, Figure S3: DVH for the planned and predicted doses, Figure S4: Cumulative dose with different reference MRIs, Figure S5: Example patient with dose mapping on MR1, MR4 and MR7, Figure S6: Dice similarity coefficients, Table S1: Literature overview of all the identified studies that investigated the change in dose, volume or migration of salivary glands during the course of radiotherapy sorted according to the publication year and alphabetically using the first author’s surname.
