# Supplementary material for: MR-Guided Adaptive Radiotherapy for Head and Neck Cancer: Prospective Evaluation of Migration and Anatomical Changes of the Major Salivary Glands
Source: Cancers (Basel). 2021 Oct 28;13(21):5404. doi: 10.3390/cancers13215404 (PMC8582485; doi:10.3390/cancers13215404)
Supplement: Supplementary file 1 [file cancers-13-05404-s001.zip › cancers-1397361-supplementary.pdf]

Supplementary Material

MR-guided adaptive radiotherapy for head-and-neck cancer: prospective evaluation of migration of the salivary glands and anatomical changes

Janita E. van Timmeren, Madalyne Chamberlain, Marta Bogowicz, Stefanie Ehrbar, Riccardo Dal Bello, Helena Garcia Schüler, Jérôme Krayenbuehl, Lotte Wilke, Nicolaus Andratschke, Matthias Guckenberger, Stephanie Tanadini-Lang, Panagiotis Balermpas

Table S1. Literature overview of all identified studies that investigated the change in dose, volume or migration of salivary glands during the course of radiotherapy, sorted according to publication year and alphabetic using the first author's surname. HNC = Head-and-neck cancer, CT = Computer tomography.

First author	Year	Patients	Imaging	Glands	Volume	Migration	Dose – compared to planning	Dose – compared to no re-planning
Barker[1]	2004	14 HNC	CT scans three times weekly	Parotids	Mean: -28.1% (0.6%/day)	Median medial shift: 3.1 mm		
Nishimura[2]	2005	33 Pharyngeal	mid-treatment CT	Parotids	Mean: -26%			
Hansen[3]	2006	13 HNC	mid-treatment CT	Parotids	Mean: -15.6-21.5%			Dmean -0.2 - -2.9 Gy
Robar[4]	2007	15 HNC	weekly CT	Parotids	Mean: -4.7% - 5.0%/week	Mean medial shift: 0.85 mm/week	Dmean: +0.2% - +2.6%	
O'Daniel[5]	2007	11 HNC	biweekly CT	Parotids			Dmean: +1.0 Gy	
Lee[6]	2008	10 HNC	daily MV CT	Parotids		Medial shift	Dmean: +15%	
Vasquez Osorio[7]	2008	10 Oropharyngeal	FU CT two weeks after end RT	Salivary glands	Mean: -5%-20%	Parotids medial shift: 1 mm<br>Submandibular glands superior		
Han[8]	2008	5 Nasopharyngeal	daily MV CT	Parotids	Mean: -40.2% (1.1%/day)		Dmedian: +2.2% per day	
Wang[9]	2009	82 HNC	mid-treatment CT scans	Salivary glands	Mean: -16.8%-26.9%			
Wu[10]	2009	11 HNC	weekly CT	Parotids	Mean: -15%		Dmean: +10%	Dmean: -8%
Bhide[11]	2010	20 HNC	weekly CT	Parotids	Mean: -35%		Dmean: +2.8 Gy	
Broggi[12]	2010	87 HNC	MV CT or kV CT at the end of treatment	Parotids	Median: -26%			
Castadot[13]	2010	10 HNC	weekly CT	Salivary glands	Mean: -0.9% --1.3%/day	Parotids mean medial shift: 3.4 mm<br>Submandibular superior shift: 1.7 mm		
Height[14]	2010	10 HNC	repeat CT after fifth week of treatment	Parotids	Median: -20.5% - -23.6%	Medial shift: 0.3 - 1.9 mm	No detectable change	
Teshima[15]	2010	20 Oral squamous cell carcinoma	CT or MR at the end of treatment	Parotids	Mean: -29%			
Wang[16]	2010	15 Nasopharyngeal	repeat CT after 18 fractions	Parotids	Median: -19.8%-20.6%	Median medial shift: 4.3-4.8 mm	Dmean: +2.57-2.97 Gy	
Houweling[17]	2011	18 Oropharyngeal cancer	FU MRI 6 weeks after end of treatment	Salivary glands	Mean: -25%			
Loo[18]	2011	5 HNC	daily MV CT	Parotids	Mean: -17.5% - -30.2%	Medial shift	Dmean: +8.9% - +19.3%	
Ricchetti[19]	2011	26 HNC	weekly CT	Salivary glands	Mean: -26.4% - -31.9%			
Beltran[20]	2012	16 HNC	repeat CT after 15 and 25 fractions	Parotids	Mean: -30% (1.5%/day)		Dmean: +4.7% - +6.1%	

Capelle[21]	2010	20 HNC	repeat CT after 15 fractions	Parotids	Mean: -17.5%			Dmean: -0.6 Gy
Fiorentino[22]	2012	10 HNC	daily CBCT	Parotids	Mean: -43.5% - -44.0% (1.8%-1.6%/day)			
Fung[23]	2012	10 Naropharyngeal	repeat kV CT after ~25 and ~35 fractions	Parotids	Mean: -32.4% - -33.3%			Dmean: -0.84 - -1.25 Gy
Lu[24]	2012	43 Naropharyngeal	repeat CT after 20 fractions	Parotids	Mean: -35.5% - -36.8%			
Hunter[25]	2013	18 Oropharyngeal	daily CBCT	Parotids	Mean: -13.3%			Dmean: -0.92 Gy
Jin[26]	2013	9 Nasopharyngeal		Parotids	Mean: -38.4% - -40.7%			Dmean: -0.31 - -0.62 Gy
Nishi[27]	2013	20 HNC	repeat CT 3-4 weeks after start RT	Parotids	Mean: -18.1%	Mean medial shift: 4.2 mm	Dmean: +5.0 Gy	
Schwartz[28]	2013	22 Oropharyngeal	daily CT	Parotids	Mean: -26%			Dmean: -0.8 - -4.1 Gy
Sanguineti[29]	2013	85 Oropharyngeal	weekly CBCT	Parotids	Mean: -30.7% - -34%			
Belli[30]	2014	46 HNC	kV CT mid-treatment and during last week of RT	Parotids	Mean: -146 mm3/day			
Fung[31]	2014	30 Naropharyngeal	two repeat CT mid-treatment	Parotids	Mean: 47.5% (1.35%/day)	Mean medial&superior shift: 1 mm/day		
Castelli[32]	2015	15 HNC	weekly CT	Parotids	Mean: -28.3%	Parotid-CTV distance -4.3 mm for 74%		Dmean: -3.6 Gy
Chitapanarux [33]	2015	17 Nasopharyngeal	repeat CT after 17 fractions	Parotids	Mean: -24.3% - -30.5%			Dmean: -1.1 Gy
Huang[34]	2015	19 Nasopharyngeal	repeat CT after each 5 fractions and at the end of treatment	Parotids	Mean: -38.0% - -39.2% (7.8%-7.9%/week)	Mean medial shift: 2.7 mm – 3.1 mm		Dmean: -3.1 - -5.6 Gy
Marzi[35]	2015	34 HNC	MRI at start, mid-treatment, and end of RT	Parotids	Mean: -31%			
Yao[36]	2015	50 Nasopharyngeal	weekly MV CT	Parotids	Median: -35% (1.07%/day)			
Dewan[37]	2016	30 HNC	repeat CT after 4 weeks of treatment	Parotids	Mean: -31.1% - -33.7%	Mean medial shift: 2.7 mm – 3.3 mm		Dmean: -5.6 Gy
Raghavan[38]	2016	6 HNC	daily MRI	Parotids	Median: -21.8% - -31.3%	Median medial shift: 0.9 mm – 1.4 mm	Dmean: +0.008 Gy – 0.01 Gy	
Zhou[39]	2016	18 Nasopharyngeal		Parotids	Mean: -26.5%			
Zhang[40]	2016	13 Oropharyngeal	weekly CT	Parotids	Mean: -28.0% - -34.5%			Dmean: -3.3 Gy
Mahmoud[41]	2017	22 HNC	repeat CT after 3 weeks and after 6 weeks of treatment	Parotids	Mean: -23.1% - -30.9%			Dmean: -9.1% - -16.4%
Zhang[42]	2017	39 Nasopharyngeal	kV CT at 10th, 20th and 30th fractions of RT	Parotids	Mean: -37.5%	Mean medial shift: 3.0 mm<br>Mean superior shift: 1.5 mm		
Hu[43]	2018	40 Nasopharyngeal	repeat CT after ~22 fractions	Parotids	Mean: -17.2% - -20%			Dmean: -0.1 Gy - -0.7 Gy

Marzi[44]	2018	40 Oropharyngeal	repeat MRI after 10 fractions and FU MRI 8 weeks after RT	Parotids	Mean: -18.2%	Medial and superior shift		
Ilangovan[45]	2020	45 HNC	repeat CT after 15 and 25 fractions	Parotids	Mean: -33.6% - -36.0%		Dmean: +4.8 Gy - +6.3 Gy	
Lee[46]	2020	64 HNC	weekly CBCT	Parotids	Mean: -25%			
Wellington dos Santos[47]	2020	49 HNC	CT at end of RT	Parotids	Mean: -20% - -21%			
Verma[48]	2020	10 HNC	repeat CT at week 3 and 5	Parotids	Mean: -33.6 - -35.6%	Mean medial shift: 2.8 mm – 3.6 mm	Dmean: +33% - +48.9%	
McDonald[49]	2021	10 HNC	daily MRI	Parotids	Range: +0.2% - -11%			Dmean: -0.50 Gy

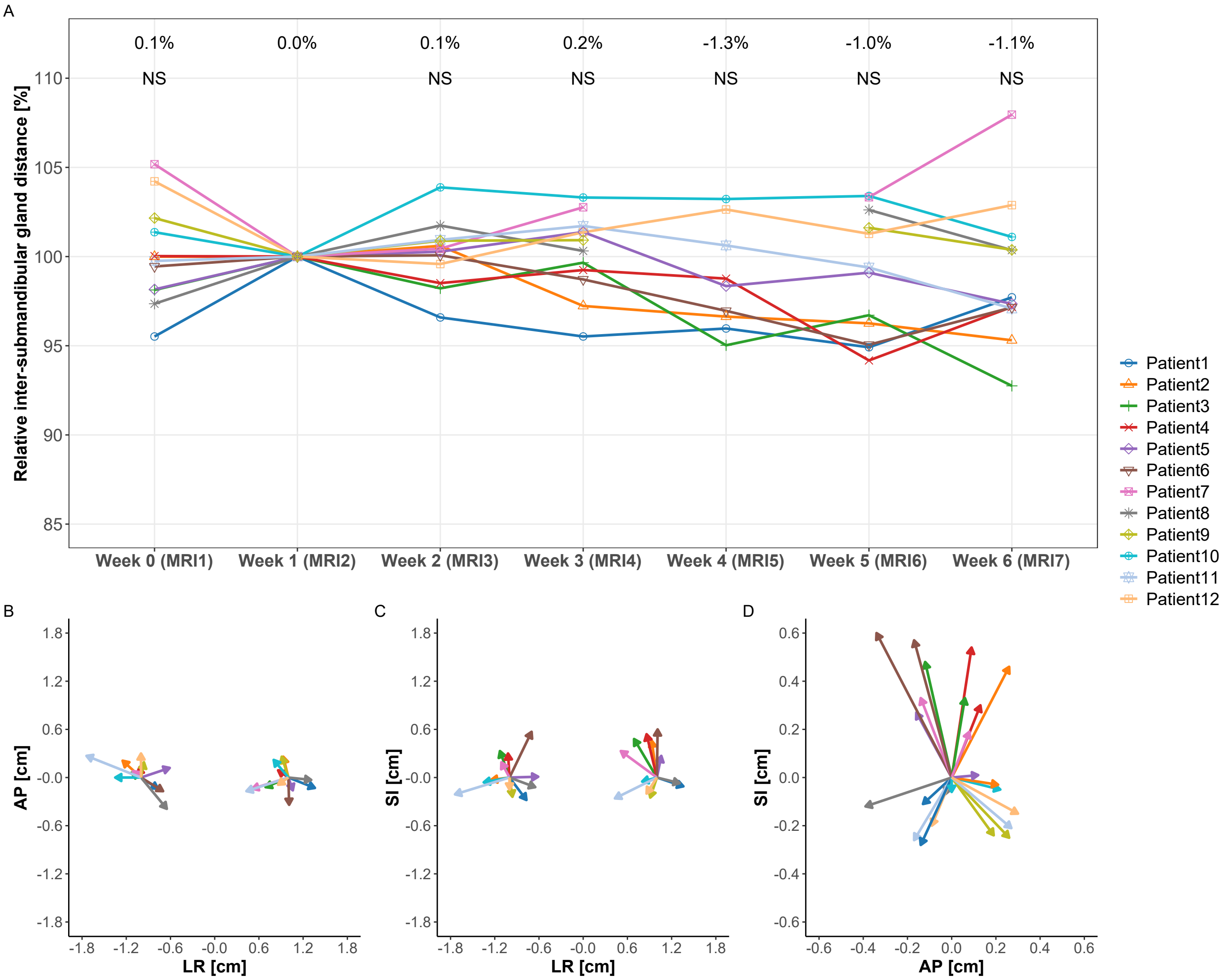

Figure S1: Migration of submandibular glands during radiotherapy. A) Change of inter-submandibular gland distance. B-D) Submandibular gland migration in AP/LR, SI/LR and SI/AP. For this representation the submandibular gland locations was corrected with the brainstem location, and then the baseline submandibular gland location was set to (-1,0) for the right submandibular gland and (1,0) for the left submandibular gland. AP = Anterior-posterior, LR = left-right, SI = superior-inferior. NS: Not significant.

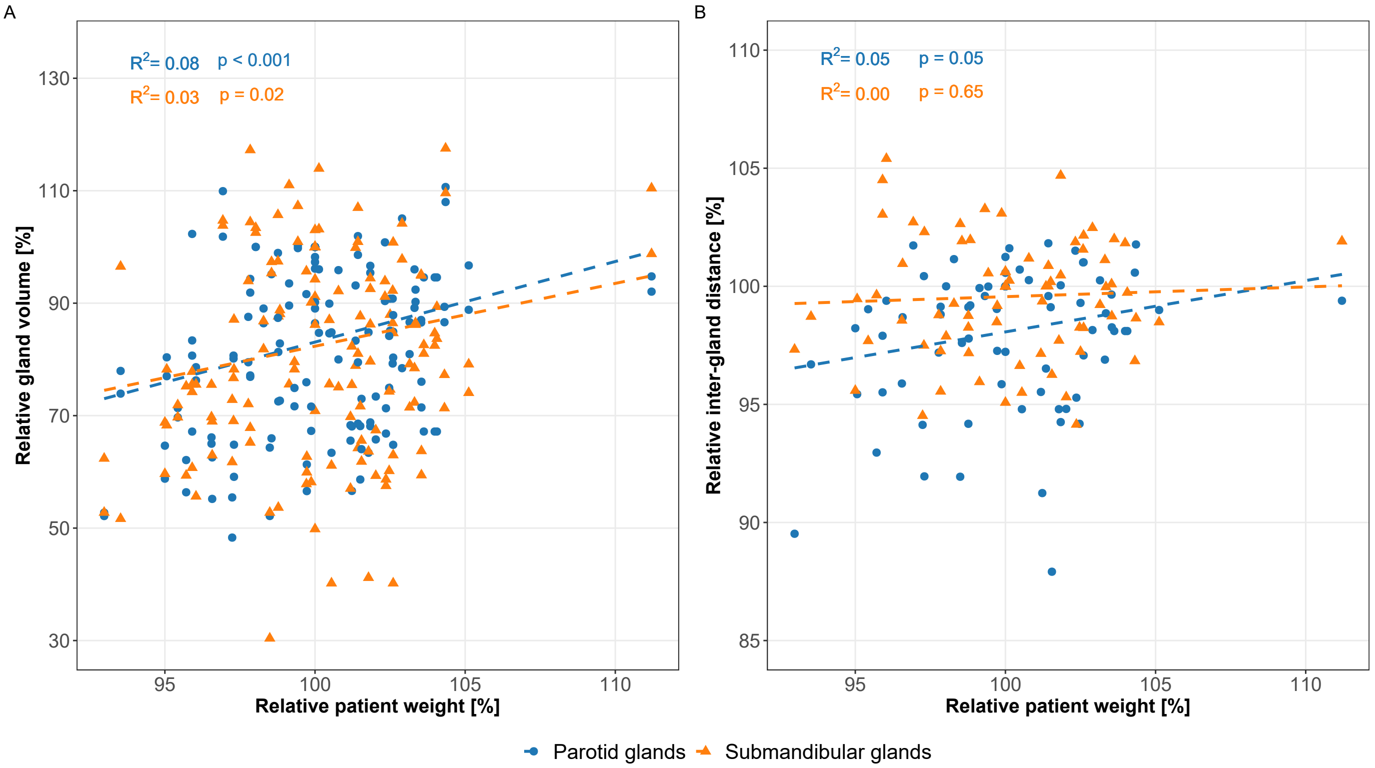

Figure S2: Scatterplot between patient weight and relative gland volume (A) or inter-gland distance (B).

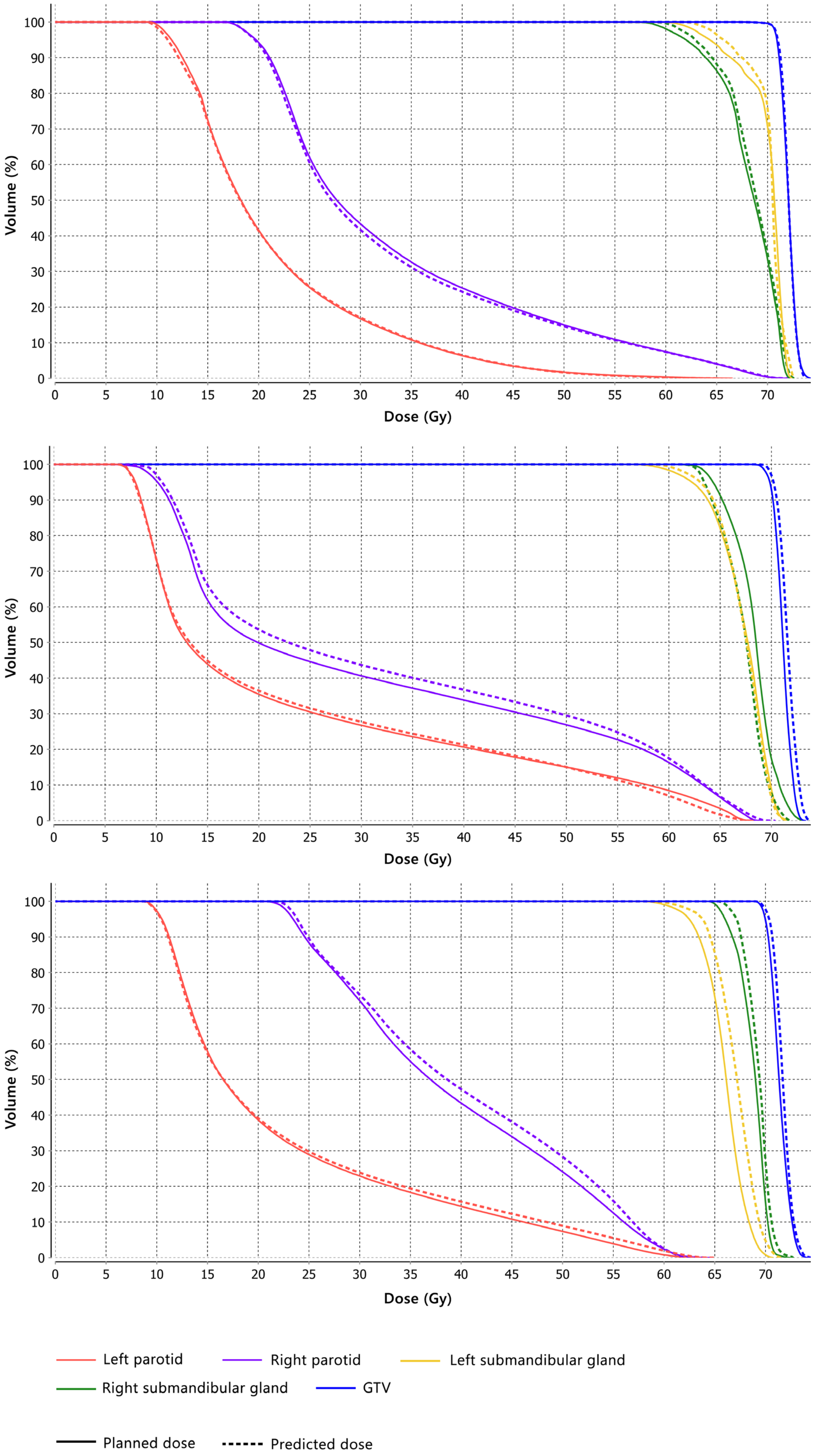

Figure S3: Cumulative-planned dose (solid lines) and cumulative-predicted dose (dashed lines) for weekly adaptations. Red is the left parotid, purple the right parotid, yellow the left submandibular gland, green the right submandibular gland, and blue the gross tumor volume (GTV).

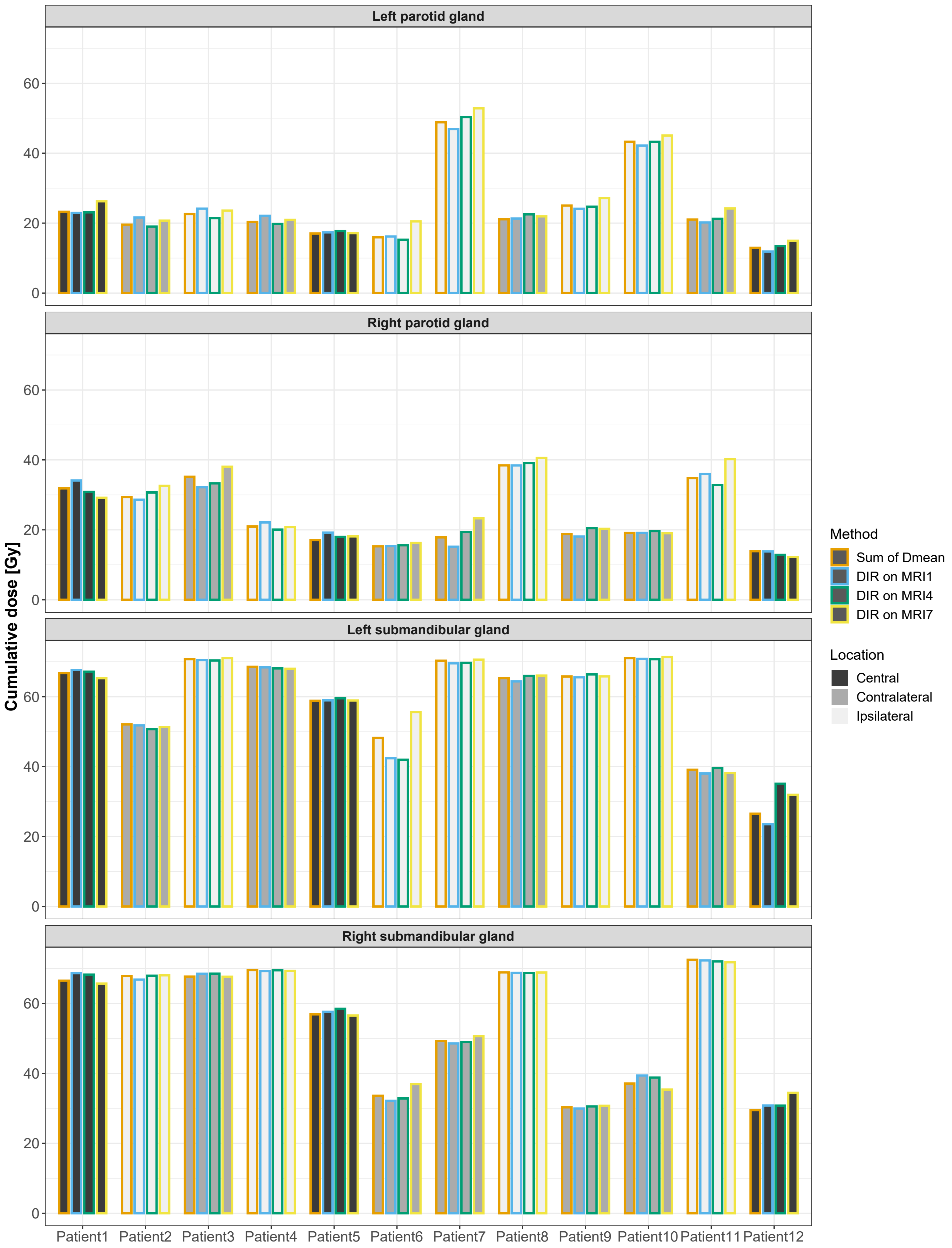

Figure S4: Differences between dose accumulations. Left) linear summation of the glands' Dmean at each time point, Second) DIR-based cumulative dose mapped onto MR1, Third) DIR-based cumulative dose mapped onto MR4 (see Figure 4) and Right) DIR-based cumulative dose mapped onto MR7. Patient 7 is shown as an example in Figure S5.

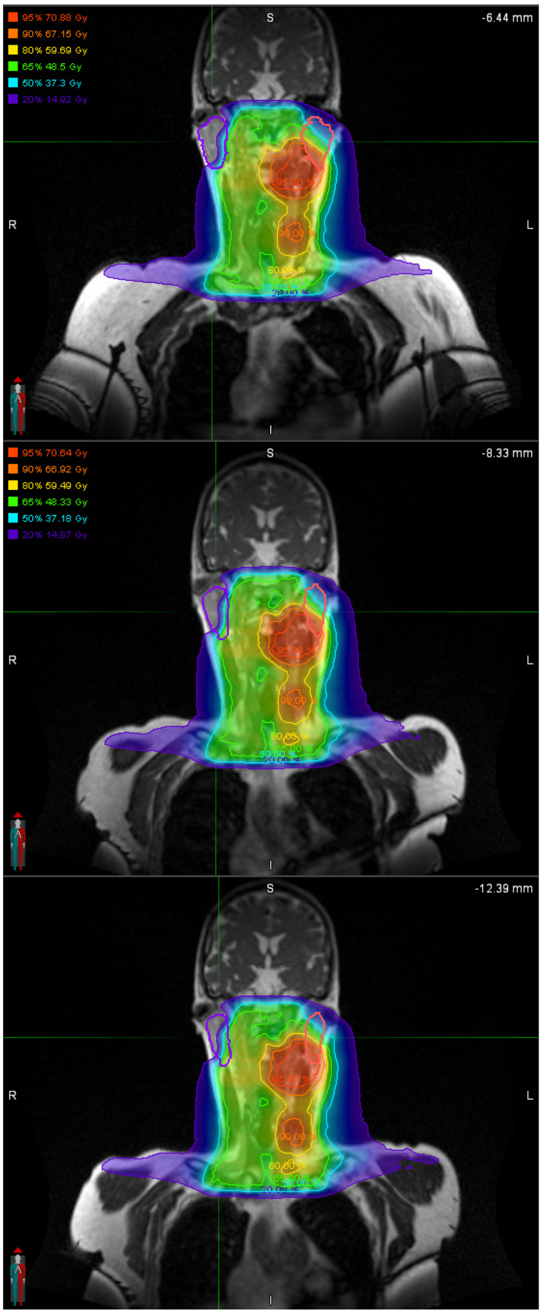

A

Dose warped onto MRI1
Right parotid Dmean = 15.2 Gy

B

Dose warped onto MRI4
Right parotid Dmean = 19.4 Gy

C

Dose warped onto MRI7
Right parotid Dmean = 23.3 Gy

Figure S5: Example patient with the DIR-based cumulative-dose mapped onto MR1 (A), MR4 (B) or MR7 (C)

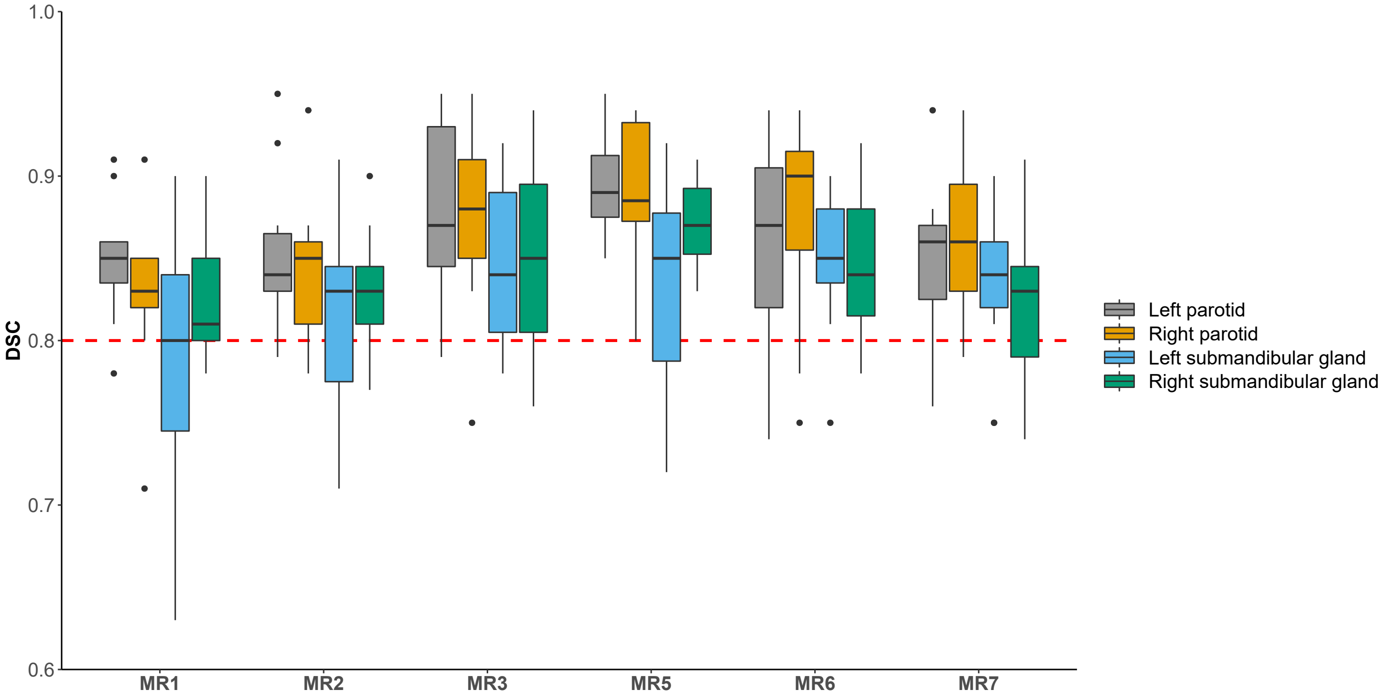

Figure S6: Dice similarity coefficient (DSC) values between the salivary glands contoured on MR4 and after deformable image registration with the remaining MRIs.

References

1. Barker, J.L., Jr.; Garden, A.S.; Ang, K.K.; O'Daniel, J.C.; Wang, H.; Court, L.E.; Morrison, W.H.; Rosenthal, D.I.; Chao, K.S.; Tucker, S.L., *et al.* Quantification of volumetric and geometric changes occurring during fractionated radiotherapy for head-and-neck cancer using an integrated ct/linear accelerator system. *Int J Radiat Oncol Biol Phys* **2004**, *59*, 960-970. https://dx.doi.org/10.1016/j.ijrobp.2003.12.024.
2. Nishimura, Y.; Nakamatsu, K.; Shibata, T.; Kanamori, S.; Koike, R.; Okumura, M.; Suzuki, M. Importance of the initial volume of parotid glands in xerostomia for patients with head and neck cancers treated with imrt. *Jpn J Clin Oncol* **2005**, *35*, 375-379. https://dx.doi.org/10.1093/jjco/hyi108.
3. Hansen, E.K.; Bucci, M.K.; Quivey, J.M.; Weinberg, V.; Xia, P. Repeat ct imaging and replanning during the course of imrt for head-and-neck cancer. *Int J Radiat Oncol Biol Phys* **2006**, *64*, 355-362. https://dx.doi.org/10.1016/j.ijrobp.2005.07.957.
4. Robar, J.L.; Day, A.; Clancey, J.; Kelly, R.; Yewondwossen, M.; Hollenhorst, H.; Rajaraman, M.; Wilke, D. Spatial and dosimetric variability of organs at risk in head-and-neck intensity-modulated radiotherapy. *Int J Radiat Oncol Biol Phys* **2007**, *68*, 1121-1130. https://dx.doi.org/10.1016/j.ijrobp.2007.01.030.
5. O'Daniel, J.C.; Garden, A.S.; Schwartz, D.L.; Wang, H.; Ang, K.K.; Ahamad, A.; Rosenthal, D.I.; Morrison, W.H.; Asper, J.A.; Zhang, L., *et al.* Parotid gland dose in intensity-modulated radiotherapy for head and neck cancer: Is what you plan what you get? *Int J Radiat Oncol Biol Phys* **2007**, *69*, 1290-1296. https://dx.doi.org/10.1016/j.ijrobp.2007.07.2345.
6. Lee, C.; Langen, K.M.; Lu, W.; Haimerl, J.; Schnarr, E.; Ruchala, K.J.; Olivera, G.H.; Meeks, S.L.; Kupelian, P.A.; Shellenberger, T.D., *et al.* Assessment of parotid gland dose changes during head and neck cancer radiotherapy using daily megavoltage computed tomography and deformable image registration. *Int J Radiat Oncol Biol Phys* **2008**, *71*, 1563-1571. https://dx.doi.org/10.1016/j.ijrobp.2008.04.013.
7. Vasquez Osorio, E.M.; Hoogeman, M.S.; Al-Mamgani, A.; Teguh, D.N.; Levendag, P.C.; Heijmen, B.J. Local anatomic changes in parotid and submandibular glands during radiotherapy for oropharynx cancer and correlation with dose, studied in detail with nonrigid registration. *Int J Radiat Oncol Biol Phys* **2008**, *70*, 875-882. https://dx.doi.org/10.1016/j.ijrobp.2007.10.063.
8. Han, C.; Chen, Y.J.; Liu, A.; Schultheiss, T.E.; Wong, J.Y. Actual dose variation of parotid glands and spinal cord for nasopharyngeal cancer patients during radiotherapy. *Int J Radiat Oncol Biol Phys* **2008**, *70*, 1256-1262. https://dx.doi.org/10.1016/j.ijrobp.2007.10.067.
9. Wang, Z.H.; Yan, C.; Zhang, Z.Y.; Zhang, C.P.; Hu, H.S.; Kirwan, J.; Mendenhall, W.M. Radiation-induced volume changes in parotid and submandibular glands in patients with head and neck cancer receiving postoperative radiotherapy: A longitudinal study. *Laryngoscope* **2009**, *119*, 1966-1974. https://dx.doi.org/10.1002/lary.20601.
10. Wu, Q.; Chi, Y.; Chen, P.Y.; Krauss, D.J.; Yan, D.; Martinez, A. Adaptive replanning strategies accounting for shrinkage in head and neck imrt. *Int J Radiat Oncol Biol Phys* **2009**, *75*, 924-932. https://dx.doi.org/10.1016/j.ijrobp.2009.04.047.
11. Bhide, S.A.; Davies, M.; Burke, K.; McNair, H.A.; Hansen, V.; Barbachano, Y.; El-Hariry, I.A.; Newbold, K.; Harrington, K.J.; Nutting, C.M. Weekly volume and dosimetric changes during chemoradiotherapy with intensity-modulated radiation therapy for head and neck cancer: A prospective observational study. *Int J Radiat Oncol Biol Phys* **2010**, *76*, 1360-1368. https://dx.doi.org/10.1016/j.ijrobp.2009.04.005.
12. Broggi, S.; Fiorino, C.; Dell'Oca, I.; Dinapoli, N.; Paiusco, M.; Muraglia, A.; Maggiulli, E.; Ricchetti, F.; Valentini, V.; Sanguineti, G., *et al.* A two-variable linear model of parotid shrinkage during imrt for head and neck cancer. *Radiother Oncol* **2010**, *94*, 206-212. https://dx.doi.org/10.1016/j.radonc.2009.12.014.
13. Castadot, P.; Geets, X.; Lee, J.A.; Christian, N.; Gregoire, V. Assessment by a deformable registration method of the volumetric and positional changes of target volumes and organs at

risk in pharyngo-laryngeal tumors treated with concomitant chemo-radiation. *Radiother Oncol* **2010**, *95*, 209-217. https://dx.doi.org/10.1016/j.radonc.2010.03.007.
14. Height, R.; Khoo, V.; Lawford, C.; Cox, J.; Joon, D.L.; Rolfo, A.; Wada, M. The dosimetric consequences of anatomic changes in head and neck radiotherapy patients. *J Med Imaging Radiat Oncol* **2010**, *54*, 497-504. https://dx.doi.org/10.1111/j.1754-9485.2010.02209.x.
15. Teshima, K.; Murakami, R.; Tomitaka, E.; Nomura, T.; Toya, R.; Hiraki, A.; Nakayama, H.; Hirai, T.; Shinohara, M.; Oya, N., *et al.* Radiation-induced parotid gland changes in oral cancer patients: Correlation between parotid volume and saliva production. *Jpn J Clin Oncol* **2010**, *40*, 42-46. https://dx.doi.org/10.1093/jjco/hyp113.
16. Wang, X.; Lu, J.; Xiong, X.; Zhu, G.; Ying, H.; He, S.; Hu, W.; Hu, C. Anatomic and dosimetric changes during the treatment course of intensity-modulated radiotherapy for locally advanced nasopharyngeal carcinoma. *Med Dosim* **2010**, *35*, 151-157. https://dx.doi.org/10.1016/j.meddos.2009.06.007.
17. Houweling, A.C.; Schakel, T.; van den Berg, C.A.; Philippens, M.E.; Roesink, J.M.; Terhaard, C.H.; Raaijmakers, C.P. Mri to quantify early radiation-induced changes in the salivary glands. *Radiother Oncol* **2011**, *100*, 386-389. https://dx.doi.org/10.1016/j.radonc.2011.08.020.
18. Loo, H.; Fairfoul, J.; Chakrabarti, A.; Dean, J.C.; Benson, R.J.; Jefferies, S.J.; Burnet, N.G. Tumour shrinkage and contour change during radiotherapy increase the dose to organs at risk but not the target volumes for head and neck cancer patients treated on the tomotherapy hiart system. *Clin Oncol (R Coll Radiol)* **2011**, *23*, 40-47. https://dx.doi.org/10.1016/j.clon.2010.09.003.
19. Ricchetti, F.; Wu, B.; McNutt, T.; Wong, J.; Forastiere, A.; Marur, S.; Starmer, H.; Sanguineti, G. Volumetric change of selected organs at risk during imrt for oropharyngeal cancer. *Int J Radiat Oncol Biol Phys* **2011**, *80*, 161-168. https://dx.doi.org/10.1016/j.ijrobp.2010.01.071.
20. Beltran, M.; Ramos, M.; Rovira, J.J.; Perez-Hoyos, S.; Sancho, M.; Puertas, E.; Benavente, S.; Ginjaume, M.; Giralt, J. Dose variations in tumor volumes and organs at risk during imrt for head-and-neck cancer. *J Appl Clin Med Phys* **2012**, *13*, 3723. https://dx.doi.org/10.1120/jacmp.v13i6.3723.
21. Capelle, L.; Mackenzie, M.; Field, C.; Parliament, M.; Ghosh, S.; Scrimger, R. Adaptive radiotherapy using helical tomotherapy for head and neck cancer in definitive and postoperative settings: Initial results. *Clin Oncol (R Coll Radiol)* **2012**, *24*, 208-215. https://dx.doi.org/10.1016/j.clon.2011.11.005.
22. Fiorentino, A.; Caivano, R.; Metallo, V.; Chiumento, C.; Cozzolino, M.; Califano, G.; Clemente, S.; Pedicini, P.; Fusco, V. Parotid gland volumetric changes during intensity-modulated radiotherapy in head and neck cancer. *Br J Radiol* **2012**, *85*, 1415-1419. https://dx.doi.org/10.1259/bjr/30678306.
23. Fung, W.W.; Wu, V.W.; Teo, P.M. Dosimetric evaluation of a three-phase adaptive radiotherapy for nasopharyngeal carcinoma using helical tomotherapy. *Med Dosim* **2012**, *37*, 92-97. https://dx.doi.org/10.1016/j.meddos.2011.01.006.
24. Lu, N.; Feng, L.C.; Cai, B.N.; Hou, J.; Wang, Y.L.; Xie, C.B. Clinical study on the changes of the tumor target volume and organs at risk in helical tomotherapy for nasopharyngeal carcinoma. *Chinese medical journal* **2012**, *125*, 87-90.
25. Hunter, K.U.; Fernandes, L.L.; Vineberg, K.A.; McShan, D.; Antonuk, A.E.; Cornwall, C.; Feng, M.; Schipper, M.J.; Balter, J.M.; Eisbruch, A. Parotid glands dose-effect relationships based on their actually delivered doses: Implications for adaptive replanning in radiation therapy of head-and-neck cancer. *Int J Radiat Oncol Biol Phys* **2013**, *87*, 676-682. https://dx.doi.org/10.1016/j.ijrobp.2013.07.040.
26. Jin, X.; Han, C.; Zhou, Y.; Yi, J.; Yan, H.; Xie, C. A modified vmat adaptive radiotherapy for nasopharyngeal cancer patients based on ct-ct image fusion. *Radiat Oncol* **2013**, *8*, 277. https://dx.doi.org/10.1186/1748-717X-8-277.
27. Nishi, T.; Nishimura, Y.; Shibata, T.; Tamura, M.; Nishigaito, N.; Okumura, M. Volume and dosimetric changes and initial clinical experience of a two-step adaptive intensity modulated

radiation therapy (imrt) scheme for head and neck cancer. *Radiother Oncol* **2013**, *106*, 85-89. https://dx.doi.org/10.1016/j.radonc.2012.11.005.
28. Schwartz, D.L.; Garden, A.S.; Shah, S.J.; Chronowski, G.; Sejpal, S.; Rosenthal, D.I.; Chen, Y.; Zhang, Y.; Zhang, L.; Wong, P.F., *et al.* Adaptive radiotherapy for head and neck cancer--dosimetric results from a prospective clinical trial. *Radiother Oncol* **2013**, *106*, 80-84. https://dx.doi.org/10.1016/j.radonc.2012.10.010.
29. Sanguineti, G.; Ricchetti, F.; Thomas, O.; Wu, B.; McNutt, T. Pattern and predictors of volumetric change of parotid glands during intensity modulated radiotherapy. *Br J Radiol* **2013**, *86*, 20130363. https://dx.doi.org/10.1259/bjr.20130363.
30. Belli, M.L.; Scalco, E.; Sanguineti, G.; Fiorino, C.; Broggi, S.; Dinapoli, N.; Ricchetti, F.; Valentini, V.; Rizzo, G.; Cattaneo, G.M. Early changes of parotid density and volume predict modifications at the end of therapy and intensity of acute xerostomia. *Strahlenther Onkol* **2014**, *190*, 1001-1007. https://dx.doi.org/10.1007/s00066-014-0669-2.
31. Fung, W.W.; Wu, V.W.; Teo, P.M. Developing an adaptive radiation therapy strategy for nasopharyngeal carcinoma. *J Radiat Res* **2014**, *55*, 293-304. https://dx.doi.org/10.1093/jrr/rrt103.
32. Castelli, J.; Simon, A.; Louvel, G.; Henry, O.; Chajon, E.; Nassef, M.; Haigron, P.; Cazoulat, G.; Ospina, J.D.; Jegoux, F., *et al.* Impact of head and neck cancer adaptive radiotherapy to spare the parotid glands and decrease the risk of xerostomia. *Radiat Oncol* **2015**, *10*, 6. https://dx.doi.org/10.1186/s13014-014-0318-z.
33. Chitapanarux, I.; Chomprasert, K.; Nobnaop, W.; Wanwilairat, S.; Tharavichitkul, E.; Jakrabhandu, S.; Onchan, W.; Traisathit, P.; Van Gestel, D. A dosimetric comparison of two-phase adaptive intensity-modulated radiotherapy for locally advanced nasopharyngeal cancer. *J Radiat Res* **2015**, *56*, 529-538. https://dx.doi.org/10.1093/jrr/rru119.
34. Huang, H.; Lu, H.; Feng, G.; Jiang, H.; Chen, J.; Cheng, J.; Pang, Q.; Lu, Z.; Gu, J.; Peng, L., *et al.* Determining appropriate timing of adaptive radiation therapy for nasopharyngeal carcinoma during intensity-modulated radiation therapy. *Radiat Oncol* **2015**, *10*, 192. https://dx.doi.org/10.1186/s13014-015-0498-1.
35. Marzi, S.; Forina, C.; Marucci, L.; Giovinazzo, G.; Giordano, C.; Piludu, F.; Landoni, V.; Spriano, G.; Vidiri, A. Early radiation-induced changes evaluated by intravoxel incoherent motion in the major salivary glands. *J Magn Reson Imaging* **2015**, *41*, 974-982. https://dx.doi.org/10.1002/jmri.24626.
36. Yao, W.R.; Xu, S.P.; Liu, B.; Cao, X.T.; Ren, G.; Du, L.; Zhou, F.G.; Feng, L.C.; Qu, B.L.; Xie, C.B., *et al.* Replanning criteria and timing definition for parotid protection-based adaptive radiation therapy in nasopharyngeal carcinoma. *Biomed Res Int* **2015**, *2015*, 476383. https://dx.doi.org/10.1155/2015/476383.
37. Dewan, A.; Sharma, S.; Dewan, A.; Srivastava, H.; Rawat, S.; Kakria, A.; Mishra, M.; T, S.; Mehrotra, K. Impact of adaptive radiotherapy on locally advanced head and neck cancer - a dosimetric and volumetric study. *Asian Pac J Cancer Prev* **2016**, *17*, 985-992. https://dx.doi.org/10.7314/apjcp.2016.17.3.985.
38. Raghavan, G.; Kishan, A.U.; Cao, M.; Chen, A.M. Anatomic and dosimetric changes in patients with head and neck cancer treated with an integrated mri-tri-(60)co teletherapy device. *Br J Radiol* **2016**, *89*, 20160624. https://dx.doi.org/10.1259/bjr.20160624.
39. Zhou, N.; Chu, C.; Dou, X.; Li, M.; Liu, S.; Zhu, L.; Liu, B.; Guo, T.; Chen, W.; He, J., *et al.* Early evaluation of irradiated parotid glands with intravoxel incoherent motion mr imaging: Correlation with dynamic contrast-enhanced mr imaging. *BMC Cancer* **2016**, *16*, 865. https://dx.doi.org/10.1186/s12885-016-2900-2.
40. Zhang, P.; Simon, A.; Rigaud, B.; Castelli, J.; Ospina Arango, J.D.; Nassef, M.; Henry, O.; Zhu, J.; Haigron, P.; Li, B., *et al.* Optimal adaptive imrt strategy to spare the parotid glands in oropharyngeal cancer. *Radiother Oncol* **2016**, *120*, 41-47. https://dx.doi.org/10.1016/j.radonc.2016.05.028.
41. Mahmoud, O.; Reis, I.M.; Samuels, M.M.; Elsayyad, N.; Bossart, E.; Both, J.; E, E.L.; Moustafa, M.; AbdAllah, M.; Takita, C. Prospective pilot study comparing the need for adaptive

radiotherapy in unresected bulky disease and in postoperative patients with head and neck cancer. *Technol Cancer Res Treat* **2017**, *16*, 1014-1021. https://dx.doi.org/10.1177/1533034617717624.
42. Zhang, Y.; Lin, C.; Wu, J.; Jiang, X.; Lee, S.W.Y.; Tam, S.Y.; Wu, V.W.C. A longitudinal evaluation of early anatomical changes of parotid gland in intensity modulated radiotherapy of nasopharyngeal carcinoma patients with parapharyngeal space involvement. *J Med Radiat Sci* **2017**, *64*, 188-194. https://dx.doi.org/10.1002/jmrs.209.
43. Hu, Y.C.; Tsai, K.W.; Lee, C.C.; Peng, N.J.; Chien, J.C.; Tseng, H.H.; Chen, P.C.; Lin, J.C.; Liu, W.S. Which nasopharyngeal cancer patients need adaptive radiotherapy? *BMC Cancer* **2018**, *18*, 1234. https://dx.doi.org/10.1186/s12885-018-5159-y.
44. Marzi, S.; Farneti, A.; Vidiri, A.; Di Giuliano, F.; Marucci, L.; Spasiano, F.; Terrenato, I.; Sanguineti, G. Radiation-induced parotid changes in oropharyngeal cancer patients: The role of early functional imaging and patient−/treatment-related factors. *Radiat Oncology* **2018**, *13*, 189. https://dx.doi.org/10.1186/s13014-018-1137-4.
45. Ilangovan, B.; Venkatraman, M.; Balasundaram, S. Volume changes during head-and-neck radiotherapy and its impact on the parotid dose - a single-institution observational study. *Journal of cancer research and therapeutics* **2020**, *16*, 575-580. https://dx.doi.org/10.4103/jcrt.JCRT_589_19.
46. Lee, D.; Zhang, P.; Nadeem, S.; Alam, S.; Jiang, J.; Caringi, A.; Allgood, N.; Aristophanous, M.; Mechalakos, J.; Hu, Y.C. Predictive dose accumulation for hn adaptive radiotherapy. *Phys Med Biol* **2020**, *65*, 235011. https://dx.doi.org/10.1088/1361-6560/abbdb8.
47. Santos, W.d.; Gomes, J.P.P.; Nussi, A.D.; Altemani, J.O.M.; Santos, M.T.B.R.D.; Hasséus, B.; Giglio, D.; Braz-Silva, P.; Costa, A.L.F.J.I.J.o.D. Morphology, volume, and density characteristics of the parotid glands before and after chemoradiation therapy in patients with head and neck tumors. **2020**, *2020*.
48. Verma, M.; Lal, P.; Maria Das, K.J.; Kumar, S. Adaptive intensity-modulated radiotherapy in head and neck cancer patients treated by chemoradiotherapy: To quantify temporal changes during the course of radiotherapy and to study the impact of these changes on the baseline treatment plans. *J Radiat Cancer Res* **2020**, *11*. https://dx.doi.org/10.4103/jrcr.jrcr_10_20.
49. McDonald, B.A.; Vedam, S.; Yang, J.; Wang, J.; Castillo, P.; Lee, B.; Sobremonte, A.; Ahmed, S.; Ding, Y.; Mohamed, A.S.R., *et al.* Initial feasibility and clinical implementation of daily mr-guided adaptive head and neck cancer radiation therapy on a 1.5t mr-linac system: Prospective r-ideal 2a/2b systematic clinical evaluation of technical innovation. *Int J Radiat Oncol Biol Phys* **2021**, *109*, 1606-1618. https://dx.doi.org/10.1016/j.ijrobp.2020.12.015.
